# Localization of Association Signal from Risk and Protective Variants in Sequencing Studies

**DOI:** 10.3389/fgene.2012.00173

**Published:** 2012-09-06

**Authors:** Abra Brisbin, Gregory D. Jenkins, Katarzyna A. Ellsworth, Liewei Wang, Brooke L. Fridley

**Affiliations:** ^1^Department of Health Sciences Research, Mayo ClinicRochester, MN, USA; ^2^Department of Mathematics, University of Wisconsin-Eau ClaireEau Claire, WI, USA; ^3^Department of Molecular Pharmacology and Experimental Therapeutics, Mayo ClinicRochester, MN, USA; ^4^Department of Biostatistics, University of Kansas Medical CenterKansas City, KS, USA

**Keywords:** rare variants, region-based analysis, multiple testing

## Abstract

Aggregating information across multiple variants in a gene or region can improve power for rare variant association testing. Power is maximized when the aggregation region contains many causal variants and few neutral variants. In this paper, we present a method for the localization of the association signal in a region using a sliding-window based approach to rare variant association testing in a region. We first introduce a novel method for analysis of rare variants, the Difference in Minor Allele Frequency test (DMAF), which allows combined analysis of common and rare variants, and makes no assumptions about the direction of effects. In whole-region analyses of simulated data with risk and protective variants, DMAF and other methods which pool data across individuals were found to outperform methods which pool data across variants. We then implement a sliding-window version of DMAF, using a step-down permutation approach to control type I error with the testing of multiple windows. In simulations, the sliding-window DMAF improved power to detect a causal sub-region, compared to applying DMAF to the whole region. Sliding-window DMAF was also effective in localizing the causal sub-region. We also applied the DMAF sliding-window approach to test for an association between response to the drug gemcitabine and variants in the gene *FKBP5* sequenced in 91 lymphoblastoid cell lines derived from white non-Hispanic individuals. The application of the sliding-window test procedure detected an association in a sub-region spanning an exon and two introns, when rare and common variants were analyzed together.

## Introduction

Traditional genome-wide association analysis approaches, which analyze a single variant at a time, are underpowered to detect associations with rare variants (Bansal et al., 2010). Small to moderate effects at multiple rare variants could play an important role in explaining the missing heritability observed for many complex traits (Manolio et al., 2009). Several proposed methods have sought to improve power by aggregating information across a set of variants, for example, in a gene (Bansal et al., 2010). Many methods assume all rare variants have the same direction of effect; such methods are subject to loss of power in the presence of both risk and protective variants. There is a need for flexible methods which can detect associations with both risk and protective variants.

Power to detect association is reduced when the region includes many non-causal variants, which decrease the signal-to-noise ratio (Li and Leal, 2008). Therefore, when causal variants are clustered in a sub-region within a larger region of interest, power would be maximized by analyzing only that sub-region, because neutral variants outside the sub-region would be excluded. However, because the location of the causal sub-region (if any) is unknown for real data, it is necessary to test multiple sub-regions. There is a need for methods which analyze sub-regions – potentially increasing power to detect association through exclusion of some neutral variants – while minimizing power loss due to multiple testing. A sliding-window based approach could meet this need. Sliding windows have been shown to improve power for detection of an association due to 1–3 causal variants, compared to single-marker analysis (Li et al., 2007; Tang et al., 2009) and haplotype block partitioning (Guo et al., 2009), even after multiple-test correction. However, there is a need for region-based sliding-window approaches to enable the analysis of longer windows containing more than three causal variants of small effect. In addition, one challenge of region-based association methods is that detecting an association in a large region is not informative about the specific functional elements within the region that may be causal. Sliding-window based analysis is a means of localizing the association signal to a smaller sub-region, such as an exon within a candidate gene or a gene within a candidate pathway.

In this paper, we present a sliding-window, region-based approach for rare variant association testing which makes no assumptions about the direction of effects. Our approach uses a novel method for analysis of rare variants, the Difference in Minor Allele Frequency test (DMAF). DMAF allows combined analysis of common and rare variants, and can be extended to the analysis of pooled sequencing data, for which many collapsing methods are not applicable. Our method allows weighting of markers based on minor allele frequency (MAF; Madsen and Browning, 2009) or functional information (Price et al., 2010). We compared DMAF with eight other methods for whole-region analysis on a comprehensive set of simulations, and found that DMAF’s use of a positive function of the difference in MAF between cases and controls is effective in retaining power across simulations involving risk and protective variants, as well as the scenarios with only risk variants. We then applied the sliding-window DMAF to simulated regions containing a cluster of approximately 65 causal variants. For a wide range of window sizes, the sliding-window approach improved power compared to the whole-region analysis and was effective in localizing the causal sub-region. Finally, we applied this method to a cell based model system to localize an association between the gene *FKBP5* and response to the drug gemcitabine.

## Materials and Methods

### DMAF rare variant testing approach

For each single nucleotide variant (SNV) *j*, *j* = 1, …, *J*, let *D_j_* represent the absolute value of the difference in MAF between cases and controls, *D_j_* = |*X_j_* − *Y_j_*|. An alternative function, *D_j_* = (*X_j_* − *Y_j_*)^2^, was also compared; these approaches are distinguished as DMAF_abs_ and DMAF_sq_. By using a positive function of the difference in MAF, our method places equal importance on risk variants (which are expected to have *X_j_* > *Y_j_*) as on protective variants (which are expected to have *X_j_* < *Y_j_*). The test statistic is then computed as the weighted sum over the variants of interest, $V=\sum_{j\in A}w_{j}D_{j}$, where *w_j_* is the weight for variant *j* and **A** is the set of variants of interest. **A** may include all variants in a window or only rare variants. We used a threshold of MAF ≤ 0.05 to classify variants as rare. When using DMAF_sq_ with equal numbers of cases and controls, the test statistic *V* is equivalent to *Q*, the test statistic of SKAT (Wu et al., 2011). Unlike SKAT, DMAF places equal weight on cases and controls, regardless of their relative sample sizes, to emphasize information available from potentially limited numbers of cases. The significance of *V* is determined empirically by permuting case-control status *n* times and recalculating *V* for each permutation. We used *n* = 1000. For the sliding-window analysis, multiple-test correction for windows of a given size was performed using a step-down approach based on a second set of permutations (see below).

Various choices exist for *w_j_*, such as weights based on functional information (Price et al., 2010). However, there are many situations in which functionally based weights are unreliable or unavailable, such as intergenic regions. For this reason, we used weights based on the MAF: $w_{j}=1∕\sqrt{n_{j}q_{j}\left(1-q_{j}\right)}$, where *n_j_* is the number of individuals genotyped (or imputed) for variant *j* and *q_j_* is the overall MAF for the variant. This model places greater emphasis on rare alleles, which are *a priori* believed to be more likely to have larger effect sizes (Manolio et al., 2009). It also prioritizes large relative differences in MAF, even for small absolute differences at rare variants. This model is similar to that used by Madsen and Browning (2009); however, we base *q_j_* on cases and controls, rather than controls only, to put equal emphasis on risk and protective alleles.

### Step-down permutation-based correction for multiple testing

For sliding-windows of a given size (number of variants), multiple-test correction was performed using a step-down permutation-based approach (Westfall et al., 1999). For each window, an empirical distribution of the test statistic *V* was generated from 1000 permutations of the phenotype. This distribution was used to produce an empirical *p*-value for the test statistic *V* for each window. The phenotype was then permuted an additional 1000 times, and an empirical *p*-value for *V* was determined for the second set of permuted phenotypes. These *p*-values comprised a *p*-value matrix **M**, consisting of *m* rows by 1000 columns, where *m* is the number of windows of the given size. The *p*-values based on the observed phenotype were then ordered from smallest to largest in the vector **p**, and the rows of **M** were reordered in the same order. Then, the first element (smallest *p*-value) of **p** was compared to ${\text{M}}_{\text{min}}=\left\{\underset{1\leq i\leq m}{\min}M_{i,j}:1\leq j\leq 1000\right\}$, the set of column minimums of **M**. The multiple-test corrected *p*-value for the window corresponding to the element **p**_1_ is the proportion of elements of **M**_min_ that are smaller than **p**_1_. The first row of **M** was then removed and **p**_2_ was compared to the column minimums of the smaller **M** to achieve the step-down correction, which is less conservative than a Bonferroni correction. To preserve monotonicity of *p*-values, the multiple-test corrected *p*-value for **p***_j_*, *j* > 1 was calculated as $\max\left(p_{j-1},\left|\left\{M_{\min}<p_{j}\right\}\right|∕1000\right)$. One goal of the current study was to assess the robustness of results to the window size. Therefore, no correction was made for the multiple window sizes tested.

### Simulation study I: Assessment of DMAF testing framework

#### Simulated data

The coalescent simulators *ms* (Hudson, 2002) and *msHOT* (Hellenthal and Stephens, 2007) were used to simulate sequence data under no natural selection for three regions. Each region was 50 kb in length and had a mutation rate of μ = 10^−8^ mutations/bp/generation, an effective population size of 10,000, and a recombination rate of 1 cM/Mb. Regions 2 and 3 also had a hotspot of length 2 kb in which the recombination rate was 15 cM/Mb. We simulated 100,000 diploid individuals and generated phenotypes according to a null model and six models with causal SNVs (Table 1). All of the models used a multiplicative model for genetic effect: Pr(*y_i_* = case|genotype) = Π*_j_ c_j_OR_ij_*, where *OR_ij_* is the odds ratio of the variants carried by individual *i* for variant *j* and *c_j_* is a constant of proportionality. For each region and genetic model, *c_j_* was chosen to produce a population prevalence of 10%. To test the sensitivity of DMAF and other methods of rare variant analysis, we sampled 100 sets of 200 cases and 200 controls from each simulated data set to mimic a small but realistic sample size for sequencing studies (Wang et al., 2010; Jeoung et al., 2012; Silva et al., 2012), in which detection of rare variant associations is more challenging than in larger studies. We included causal effects at both rare and low-frequency variants to permit sufficient power for discrimination among analysis methods using realistic effect sizes for a sample size of 400 subjects.

**Table 1:** **Summary of models used in Simulation study I and II**.

Simulation study	Model	Type of causal variants	MAF of causal variants
I (Entire region)	A	Risk	≤0.05
	B	Risk	≤0.04
	C	Risk	≤0.06
	D	Risk and Protective	≤0.05
	E	Risk	≤0.05, 0.10
	F	Risk and Protective	≤0.05, 0.10
II (Sub-Region)	G	Risk	≤0.05
	H	Risk and Protective	≤0.05

In models A–F, half of the rare variants were risk alleles, and half were neutral or protective; different thresholds were used to classify variants as rare (Table 1). The DMAF method is expected to have greater power for models D and F than A and E, respectively, because models D and F contain more causal variants. In contrast, methods that do not accommodate protective variants are expected to have reduced power for models D and F, as the signal from risk variants will be canceled out by protective variants in the same region. Like previous simulations for rare variant analysis (Liu and Leal, 2010; Neale et al., 2011), we applied smaller ORs for more common alleles: OR = 1.5 for risk variants with MAF > 0.01, OR = 1.7 for 0.001 ≤ MAF ≤ 0.01; OR = 2.0 for MAF < 0.001. The ORs for protective variants were the reciprocals of the effect sizes for the risk variants. In contrast to many previous simulations, we have simulated large regions with many causal variants (Table 2). Due to the low MAF of the causal variants and the low population prevalence, the proportion of trait variance (Nagelkerke, 1991) explained by the set of causal variants ranged from 3.8 to 13.6%, with a mean of 7.9%. These values are consistent with the proportion of variation explained by individual linkage groups for growth-related phenotypes in brook charr (Sauvage et al., 2012) and the proportion explained by the set of known loci for type 2 diabetes in humans (Taneera et al., 2012). Therefore, our simulations realistically model complex traits influenced by a large number of variants of small effect, a situation that has been observed and hypothesized in humans and other species (Manolio et al., 2009; Ai et al., 2012; Marian, 2012).

**Table 2:** **Number of risk (protective) variants per region in each simulation model**.

Model	Region 1 (262 variants)	Region 2 (237 variants)	Region 3 (233 variants)
A	100 (0)	96 (0)	100 (0)
B	97 (0)	95 (0)	98 (0)
C	105 (0)	97 (0)	102 (0)
D	100 (100)	96 (95)	100 (99)
E	101 (0)	97 (0)	101 (0)
F	101 (100)	97 (95)	101 (99)
G	67 (0)	64 (0)	67 (0)
H	34 (33)	32 (32)	34 (34)

#### Rare variant association methods assessed

We compared DMAF to eight other rare variant association testing methods (Table 3). Both DMAF_abs_ and DMAF_sq_ were used to analyze all variants or variants with MAF ≤ 0.05, denoted as DMAF_abs,all_, DMAF_sq,all_, DMAF_abs,rare_, or DMAF_sq,rare_, respectively. The C-alpha test, like DMAF, was applied to all variants or to rare variants only (MAF ≤ 0.05), denoted as C-alpha_all_ or C-alpha_rare_. All other methods, except Variable Threshold (VT), were applied to variants with MAF ≤ 0.05. KBAC was applied using software obtained from the authors; VT was applied using software obtained^1^. All other approaches were implemented in R (R Development Core Team, 2011).

**Table 3:** **Rare variant association methods**.

Method	First author, reference	Protective	Pooling	Implementation
DMAF	Brisbin	Y	Subjects	
CMC	Li (Li and Leal, 2008)	N	SNVs, then Subjects	SNVs with MAF ≤ 0.01 collapsed (default); variants with MAF > 0.01 analyzed with Hotelling T^2^
RVT1	Morris (Morris and Zeggini, 2010)	N	SNVs	Logistic regression
KBAC	Liu (Liu and Leal, 2010)	N	SNVs	Default
WSS	Madsen (Madsen and Browning, 2009)	N	SNVs	Empirical *p*-value from 500 permutations
VT	Price (Price et al., 2010)	N	SNVs	10,000 permutations, variant weights = 1
Hotel	Hotelling (Hotelling, 1931), Xiong (Xiong et al., 2002)	Y	Subjects	Blocks of 10 SNVs were analyzed with *manova*, combined with Fisher’s method
aSum	Han (Han and Pan, 2010)	Y	SNVs	Empirical *p*-value from 500 permutations; α_0_ = 0.1 (default)
C-alpha	Neale (Neale et al., 2011)	Y	Subjects	Empirical *p*-value from 500 permutations; singletons pooled

*“Protective” column indicates whether the method is designed to accommodate protective variants; “pooling” indicates the dimension across which information is pooled*.

The nine rare variant association testing approaches differed in the dimension across which information is pooled. For example, DMAF computes the frequency difference for each variant; thus information from all subjects is pooled into a single piece of information for the variant. In addition to DMAF, the methods C-alpha, Hotelling’s T^2^, and CMC also pool information across subjects. In contrast, the other methods examined pool information across variants.

### Simulation study II: Sliding-window DMAF analysis

Using the sequence data from simulation study I, we simulated two sets of phenotypes with causal variants clustered in a sub-region of regions 1, 2, and 3. In both models, the set of rare variants (MAF ≤ 0.05) was subdivided into thirds based on position. The first and last third of rare variants and all common variants were neutral. The middle third of rare variants were all risk variants (model G) or half risk, half protective (model H). The effect sizes for risk variants were OR = 1.7 for variants with MAF > 0.01, OR = 2.0 for 0.001 ≤ MAF ≤ 0.01, and OR = 2.2 for MAF < 0.001. We analyzed 1000 simulated data sets based on models G and H for regions 1, 2, and 3 using window sizes ranging from 10 SNVs to the entire region, in increments of 10 SNVs. For the longest window sizes, fewer than 1000 simulations were analyzed since not all simulations included more than 110 polymorphic variants. Window sizes that were analyzed for 700 or more simulations were included in the power calculations.

While models G and H included effects at rare variants only, both rare and common variants were analyzed together to reflect realistic circumstances under which the MAF threshold for causal “rare” variants is unknown. DMAF_abs_ was used for the analysis since it outperformed DMAF_sq_ for a majority of the scenarios in simulation study I when rare and common variants were analyzed together. The window position was shifted in increments of 5 SNVs or 10% of the window length, whichever was greater. In addition to the analysis of models G and H, we analyzed 1000 simulations based on a null model for each region and window size to check that type I error was controlled.

An analysis for a given simulation and window size was considered to have detected an association if the multiple-test corrected *p*-value for any window was less than 0.05. In addition to assessing power, we were interested in the effectiveness of sliding windows for localizing the causal sub-region. For each simulation and window size, we determined whether the window (or set of windows) with the most significant *p*-value overlapped the causal sub-region by at least half the length of the window or set (measured by the number of markers).

### Application of DMAF to a pharmacogenomic study

Gemcitabine is a chemotherapy drug used to treat pancreatic, breast, and other solid tumors. A previous expression study identified *FKBP5* as a candidate gene for association with gemcitabine resistance (Li et al., 2008). Using Illumina’s Genome Analyzer, we resequenced *FKBP5* in 91 lymphoblastoid cell lines derived from Caucasians in the Human Variation Panel (Li et al., 2008), and identified 641 variants. The quantitative drug response phenotype of gemcitabine IC50 (effective dose that kills 50% of the cells) was estimated using a four parameter logistic model per cell line (Gallant, 1987), followed by the adjustment of log(IC50) for sex, age, and batch of cell lines (Tan et al., 2011). We defined a binary endpoint, with the top 50% of adjusted log IC50 values considered “resistant,” and the bottom 50% considered “sensitive.” All SNVs in *FKBP5* were analyzed using DMAF and the other methods listed in Table 3. Subsequently, a sliding-window analysis was performed using DMAF in windows of length 10–50 SNVs, with window size and position adjusted in increments of 5 SNVs.

## Results

### Simulation study I: Assessment of DMAF testing framework

**Figure 1:**
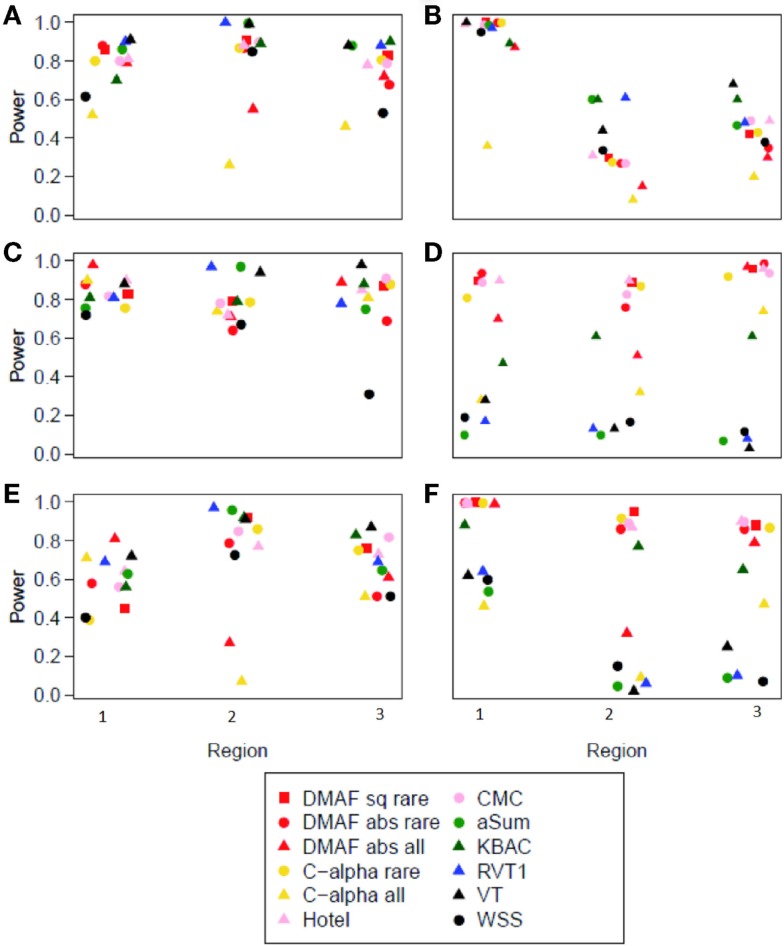
**Power at empirical type I error = 0.05 for each method and region**. **(A–F)** show results from corresponding models **(A–F)**.

The difference in Minor Allele Frequency test, along with C-alpha, CMC, and aSum, had slightly elevated type I error rates (Table 4). For a fair comparison across methods, we computed the power of each method at an empirical type I error rate of 0.05. When applied to simulations without protective variants (models A, B, C, and E), most methods performed well (Figure 1), with WSS and methods analyzing all variants having lower power. In the presence of both risk and protective variants (models D and F), the methods DMAF, C-alpha, Hotelling’s T^2^, and CMC (warm colors, Figures 1D,F) had greater power than other methods. This demonstrates that DMAF is as powerful as or more powerful than a wide range of frequently used methods, establishing its feasibility as the base method for a sliding-window analysis approach. As expected, the methods KBAC, RVT1, VT, and WSS, which assume that all causal variants have the same direction of effect, suffered reduced power on models D and F compared to models A and E, respectively, while DMAF, C-alpha, and Hotelling’s T^2^ experienced increased power or no significant change in power (Table 5). CMC did not suffer reduced power, although it is not specifically designed to accommodate protective variants, while aSum suffered reduced power despite its intended accommodation of protective variants.

**Table 4:** **Type I error rates for rare variant association methods**.

Method	Type I error rate
DMAF sq rare	0.067
DMAF abs rare	0.057
DMAF abs all	0.053
C-alpha rare	0.067
C-alpha all	0.053
Hotel	0.020
CMC	0.067
aSum	0.053
KBAC	0.030
RVT1	0.043
VT	0.040
WSS	0.037

*Type I error rate calculated for null simulations across regions 1, 2, and 3 at a nominal α = 0.05*.

**Table 5:** **Difference in power of each method between models D and A and models F and E**.

Region	Models	DMAF sq rare	DMAF abs rare	DMAF abs all	C-alpha rare	C-alpha all	Hotel	CMC	aSum	KBAC	RVT1	Price	WSS
1	D–A	**0.04**	**0.06**	−0.09	**0.01**	−0.24	**0.09**	**0.09**	−0.76	−0.23	−0.73	−0.63	−0.43
1	F–E	**0.55**	**0.42**	**0.18**	**0.61**	−0.25	**0.35**	**0.44**	−0.09	**0.32**	−0.05	−0.1	**0.2**
2	D–A	−0.02	−0.11	−0.04	0	**0.06**	**0.02**	−0.07	−0.9	−0.28	−0.87	−0.86	−0.68
2	F–E	**0.03**	**0.07**	**0.05**	**0.06**	**0.02**	**0.1**	**0.04**	−0.91	−0.15	−0.91	−0.89	−0.58
3	D–A	**0.13**	**0.31**	**0.25**	**0.11**	**0.28**	**0.18**	**0.15**	−0.81	−0.29	−0.8	−0.85	−0.41
3	F–E	**0.12**	**0.35**	**0.18**	**0.12**	−0.04	**0.17**	**0.08**	−0.56	−0.18	−0.59	−0.62	−0.44

*Positive values (bold) indicate increased power when protective variants are added to the simulations*.

Models A, B, and D had no causal variants with MAF > 0.05. For these simulations, DMAF_rare_ consistently outperformed DMAF_all_. This is consistent with previous findings that including neutral variants in the analysis decreases power (Li and Leal, 2008). Models C, E, and F included causal variants with MAF > 0.05. For these models, DMAF_all_ was not consistently superior to DMAF_rare_ (Figure 1). This contrasts with the results of Li and Leal (2008), who found that excluding causal variants from analysis was more detrimental to power than including excess neutral variants. When rare and common variants were analyzed together, power for DMAF_abs_ was better than or equal to DMAF_sq_ in 15 out of 18 scenarios. For this reason, DMAF_abs_ was used for the analysis of Simulation study II.

### Simulation study II: Sliding-window DMAF analysis

**Figure 2:**
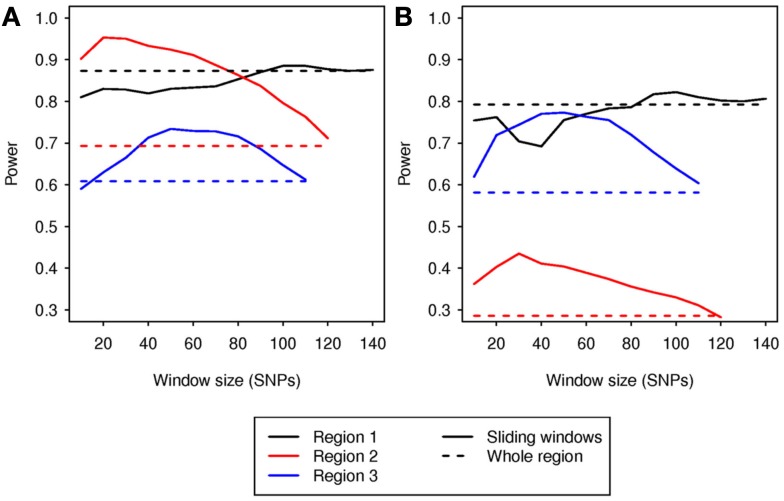
**Power vs. sliding window size**. Solid lines depict power for each window size; dashed lines indicate power for analysis of the whole region, without sliding windows for **(A)** model G and **(B)** model H. Power is for a nominal α = 0.05.

At α = 0.05, the type I error rates for the sliding-window analyses were between 0.037 and 0.058 for all window sizes that allowed analysis of at least 700 simulations. Therefore, the step-down permutation procedure adequately controlled type I error. For region 1, most sizes of sliding window gave power similar to the whole-region analysis. For regions 2 and 3, the sliding-window analysis outperformed the whole-region analysis for all window sizes from 30 to 110 SNVs (Figure 2). This demonstrates that sliding-window DMAF can improve power compared to a whole-region analysis, and the improvement is robust to choice of window size. The sliding-window approach was also effective in localizing the causal sub-region. The window with the most significant *p*-value overlapped the causal sub-region by at least half the window’s length in a majority of simulations (Table 6), particularly for regions 2 and 3.

**Table 6:** **Fraction of simulations in which the window or set of windows with the most significant *p*-value overlapped the causal sub-region by more than half the window or set’s length**.

Window size	Region and model					
	1G	1H	2G	2H	3G	3H
10	0.735	0.614	0.942	0.650	0.848	0.906
20	0.704	0.585	0.959	0.715	0.859	0.948
30	0.559	0.548	0.931	0.728	0.882	0.940
40	0.419	0.486	0.868	0.723	0.866	0.920
50	0.378	0.422	0.643	0.718	0.800	0.860
60	0.349	0.367	0.554	0.621	0.755	0.775
70	0.395	0.373	0.485	0.546	0.688	0.667
80	0.484	0.569	0.297	0.158	0.615	0.569

### Application of association methods to a pharmacogenomic study

When rare variants alone were analyzed, no method found a significant association (*p* < 0.05) between *FKBP5* and gemcitabine sensitivity (Table 7). When rare and common variants were analyzed together, we found a significant association using DMAF, along with CMC, Hotelling’s T^2^, and C-alpha*, an alternative version of C-alpha based on heterogeneity of odds ratios (Zelterman and Chen, 1988). This suggests that variants with MAF > 0.05 play an important role in the detection of gemcitabine response associations, at least when sample sizes are small. This possibility is supported by the fact that the VT method, which utilizes multiple MAF thresholds, had the next lowest *p*-value (0.129). The methods aSum, KBAC, RVT1, and WSS were unable to detect a significant association even when common variants were included in the analysis.

**Table 7:** ***P*-values for association between gemcitabine sensitivity and genotypes at 641 loci in *FKBP5***.

Method	*P*-value	
	Rare variants	All variants
DMAF_sq_	0.511	**0.030**
DMAF_abs_	0.439	**0.049**
VT	NA	0.129
C-alpha	0.686	0.252
C-alpha*	0.675	**0.005**
aSum	0.300	0.146
CMC	0.700	**0.007**
Hotel	0.757	**7.12E-05**
KBAC	0.414	0.301
RVT1	0.303	0.168
WSS	0.472	0.449

P-values less than 0.05 are in bold. *Indicates modified version based on heterogeneity of odds ratios.

Despite the implication of an association with common variants, a single-marker analysis of the 152 SNVs with MAF > 0.05 found no variants significant at the α = 0.05 level after correction for multiple testing. This demonstrates the value of methods which aggregate information from multiple variants even when common variants may be associated with the trait.

**Figure 3:**
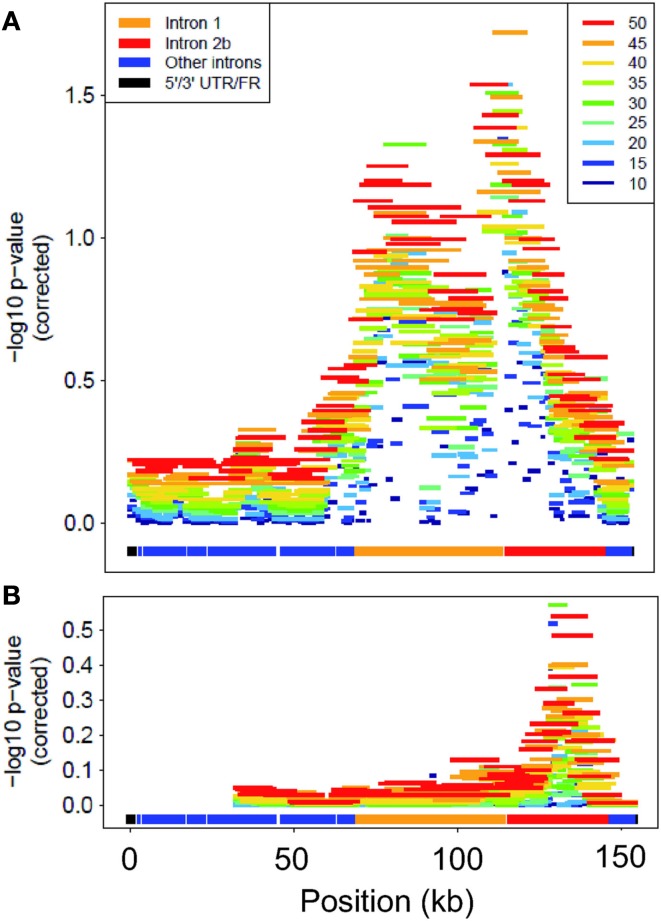
**Corrected-log10 *p*-value for each window in FKBP5**. Colors differentiate lengths of windows for analysis of **(A)** all variants and **(B)** only rare variants. “Position” is kb from start of gene.

The sliding-window analysis of *FKBP5* successfully localized the association peak to the sub-region spanning introns 1 and 2b and the exon between them (Figure 3). All window lengths identified a peak in this region, further demonstrating our method’s robustness to window size. Analysis using only rare variants localized the peak to approximately the same location, intron 2b; however, this association was not significant after multiple-test correction.

## Discussion

In this paper, we introduced DMAF, a novel approach for aggregating information across a genetic region to increase power for association testing. DMAF’s use of a positive function of the difference in minor allele frequencies between cases and controls places equal importance on risk and protective variants, and its accommodation of both rare and common variants, with weights based on MAF or biological information (e.g., functional variants given more weight), gives our method flexibility to adapt to a wide range of traits. DMAF demonstrates improved power compared to many widely used methods in the presence of both risk and protective variants. Its sliding-window implementation can increase power to detect an association due to a causal sub-region relative to whole-region analysis, and can localize associations within a gene or region. Both implementations of DMAF are available as an R package^2^.

On simulations with both risk and protective variants, rare variant analysis methods which pooled data from individuals (DMAF, CMC, Hotelling’s T^2^, C-alpha) had the greatest power. We also found that aSum, a method designed to accommodate protective variants but which pools information from variants, had weaker power on simulations with protective variants. Taken together, these results suggest that pooling data from individuals improves power for analysis of risk and protective rare variants. This indicates that sequencing pooled DNA could be used as a cost-saving measure in association studies without loss of power due to a combination of risk and protective variants.

Our simulations of causal variants clustered in a sub-region demonstrate that by using sliding windows, the power gained from reducing the proportion of neutral variants in windows which overlap the causal sub-region can outweigh the power loss due to multiple testing of the various window locations. The superior performance of the sliding-window approach on regions 2 and 3, compared to region 1, could be due to the hotspots in these regions, which interrupt linkage disequilibrium (LD) between the causal sub-region and the rest of the region. This reduced correlation results in a lower signal-to-noise ratio (compared to region 1) when non-causal variants are included in the analysis. The fact that a wide range of window sizes in the sliding-window analysis performed better than the whole-region analysis demonstrates that the sliding-window approach is a robust, powerful alternative for region-based rare variant analysis.

In the sliding-window analysis of *FKBP5* for gemcitabine response, the strongest association was *p* = 0.019, which was more significant than the whole-gene association (*p* = 0.049); however, multiple tests (window sizes) were required to achieve this. Due to the robustness of results to the window size that was demonstrated in our simulations and real data analysis, in the future it would be possible to perform a sliding-window analysis using a single window size. This could result in increased power, without the need for additional multiple-test correction. Sliding-window analyses also allow localization of region-based association signals, which will be valuable for understanding the functional regions responsible for identified associations.

To highlight the usefulness of region-based association methods such as DMAF, our simulation studies involved large numbers of causal variants which explain a modest proportion of the trait variance. This is a realistic model for sequencing studies of candidate genes or pathways. In a candidate gene study, many non-synonymous changes to the gene could affect the shape of the protein encoded by the gene, and many synonymous variants could each have a small impact on the protein’s translation rate. However, if these variants are rare SNVs, or even *de novo* mutations, then a region-based analysis method such as DMAF would be necessary to detect the variants’ combined effect on a trait influenced by the protein. In candidate pathway studies, multiple genes are grouped to detect their combined effect on a trait. If each gene in the pathway contributes a few rare variants of moderate effect, then the combined whole-region analysis would include dozens or hundreds of causal variants, as simulated here.

The results of our simulation studies (Table 6) indicate that the sliding-window analysis is effective in localizing associations within a region using a relatively small sample size of 200 cases and 200 controls. This localization may implicate a fairly large sub-region, as in the gemcitabine analysis of 91 cell lines, in which the association peak spanned two introns comprising approximately 77 kb. In the future, it would be valuable to analyze a larger sample, as this might enable more precise localization.

The dichotomization of gemcitabine response based on upper and lower 50% of IC50 values is somewhat artificial, as it results in assigning distinct phenotypes to individuals with similar intermediate responses. DMAF, like many methods for rare variant analysis, is currently applicable only to binary traits. In the future, it would be beneficial to extend DMAF to quantitative traits. It would also be worthwhile to explore the possibility of determining window size based on number of base pairs or extent of LD, rather than number of SNVs. Finally, it would be valuable to explore other variant-weighting schemes, such as a positional weighting scheme within each sliding window.

## Conflict of Interest Statement

The authors declare that the research was conducted in the absence of any commercial or financial relationships that could be construed as a potential conflict of interest.

## Footnotes

^1^http://genetics.bwh.harvard.edu/rare_variants

^2^http://mayoresearch.mayo.edu/fridley_lab/software.cfm
